# Exploring the relationship between autophagy and Gefitinib resistance in NSCLC by silencing *PDLIM5* using ultrasound-targeted microbubble destruction technology

**DOI:** 10.1186/s12935-022-02718-4

**Published:** 2022-09-25

**Authors:** Yao Zhang, Wenhao Lv, Hui Li, Tiantian Dong, Hao Wu, Chunhong Su, Hong Shu, Fang Nie

**Affiliations:** 1 Department of Ultrasound Diagnosis, Lanzhou University Second Hospital, Lanzhou University, Lanzhou, China; 2 Department of Emergency, Lanzhou University Second Hospital, Lanzhou University, Lanzhou, China

**Keywords:** Ultrasound-targeted microbubble destruction, *PDLIM5*, Gene silencing, Nanobubbles, Non-small cell lung cancer

## Abstract

**Background:**

Ultrasound-targeted microbubble destruction (UTMD) technology is a new drug and gene delivery strategy. This study investigates novel ultrasound (US) sensitive siRNA-loaded nanobubbles (siRNA-NBs) to explore the relationship between PDLIM5 mediated autophagy and drug resistance development using epidermal growth factor tyrosine kinase inhibitors (EGFR-TKIs) in the treatment of non-small cell lung cancer (NSCLC).

**Methods:**

US sensitive siRNA-NBs were designed to inhibit the expression of *PDLIM5* in gefitinib-resistant human NSCLC PC9GR cells in vitro. The expression of autophagy-related proteins (P62 and LC3-II/I) and autophagosomes in PC9GR cells after *PDLIM5* gene silencing were explored.

**Results:**

US-sensitive *PDLIM5*-targeted siRNA-NBs were effectively delivered into PC9GR cells, inhibiting *PDLIM5* expression, increasing LC3-II/I and p62 expressions and increasing autophagosomes in PC9GR cells in vitro.

**Conclusions:**

Using UTMD, US-sensitive siRNA-NBs have the potential as an ideal delivery vector to mediate highly effective RNA interference for NSCLC cells. Furthermore, PDLIM5 plays a role in the autophagy-mediated resistance in gefitinib-resistant PC9GR cells.

## Background

Human lung cancer is the most malignant and highly invasive of human tumours and the leading cause of cancer-related death worldwide, with an estimated 2 million new cases and 176 million deaths annually [1]. In 2022, there will be approximately 4,820,000 new cancer cases and 3,210,000 cancer deaths in China [2]. The most common cancers are lung cancer, which is the leading cause of cancer death in China [2]. Moreover, the prognosis of individuals diagnosed with advanced cancer is worse than those diagnosed at the early stage. World Health Organisation divides lung cancer into small cell lung cancer and NSCLC, which accounts for 85% of all lung cancers. Lung adenocarcinoma (LUAD) is a histological category of NSCLC [3]. In the past decade, molecular targeted therapy has drastically changed the treatment mode of NSCLC. Although targeted therapies have shown improved outcomes in patients with NSCLC, their treatment response is often incomplete and temporary [1]. Resistance to targeted drugs can be divided into intrinsic, adaptive and acquired drug resistances [4]. Some tumours exhibit intrinsic resistance and do not respond to initial treatment, which could be related to treatment-insensitive driver mutations. In such cases, EGFR-TKIs, gefitinib /erlotinib, are recommended as the first line of treatment for patients with EGFR mutations; however, they do not significantly improve the overall survival, owing to the intrinsic resistance developed during treatment as seen in patients with NSCLC.

Recently, the abnormal intracellular autophagy regulation mechanism has been associated with various diseases. The role of autophagy in tumour development and its role in anti-tumour drug functionality are current popular topics of research. While, the role of autophagy in cancer is controversial and no consensus has been reached regarding the precise involvement of autophagy in cancer progression and inhibition [5]. When autophagy exerts a pro-survival role, its stimulation results in a decrease in apoptosis of cancer cells, paving the way for cancer progression [6]. However, when autophagy possesses an anti-tumor role, its activation significantly enhances sensitivity of cancer cells to therapy [5]. In primary lung tumors, autophagy associated with drug resistance has been reported as a biomarker for subclassification, differentiation, and local metastasis [7]. Therefore, in order to develop a novel therapeutic regimen for cancer, the exact role of autophagy should be defined and, based on its role, an inhibitor or activator be recommended. It is worth mentioning that the type of autophagy taking place, either tumor-promoting or tumor-suppressing, determines the response of cancer cells to therapy [7, 8]. Hence, the exact role of autophagy in cancer should be determined before regulatory methods are explored [9–12]. For the reason mentioned above, exploring the mechanism of autophagy in the therapeutic resistance of EGFR-TKIs is important to overcome the drug resistance to EGFR-TKIs and improve the clinical treatment of NSCLC.

Owing to its dual role, autophagy can either suppress or induce chemoresistance [13]. Furthermore, high autophagic flux in cancer has been associated with chemotherapy resistance and poor prognosis in patients with cancer [14]. In order words, cancer cell sensitivity to chemotherapeutic drugs can be enhanced by inhibiting autophagy. Clinical studies have shown that the inhibition of autophagy enhances the resistance of gastric cancer [15], prostate cancer [16], ovarian carcinoma [17], hepatocellular carcinoma [18], colorectal cancer [14] and osteosarcoma [19] to chemotherapy. Conversely,, literature also reports that the activation of autophagy enhances the sensitivity of tumour cells to drugs or activation of the autophagy signalling pathway increases the level of autophagy to improve drug sensitivity via promoting apoptosis [5]. Similarly, there is also evidence demonstrating that autophagy induction participates in elevated efficacy of chemotherapy in cancer elimination [20–22].

PDLIM5, also known as Enigma homolog (ENH), is a member of the PDZ LIM family of proteins, containing one PDZ domain and three LIM domains [23, 24]. PDLIM5, a scaffold protein, has been reported to be involved in the signalling regulation of membrane-related proteins, cytoskeletal proteins and various signalling molecules along with the progression of various tumours [4]. Its expression level increases in NSCLC and high PDLIM5 protein expression in tumour tissues is closely related to NSCLC progression and poor prognosis. Moreover, recent studies have shown that PDLIM5 could participate in cell autophagy by regulating NSCLC drug resistance, helping cells adapt to the stressful environment and promoting cell survival, which leads to tumour drug resistance development [4]. Therefore, the sensitivity of tumour cells to EGFR-TKIs is speculated to improve in NSCLC-resistant cells by affecting the effect of autophagy on epidermal growth factor receptor (EGFR) and reducing the resistance of EGFR mutant NSCLC cells. Thus, this approach has potential as a new means of treating drug-resistant NSCLC.

US has long been medically used and is one of the most important diagnostic tools in clinical practice due to its non-invasiveness, radiological safety, low price and high repetitive nature [25]. UTMD technology is a recent and novel drug and gene delivery strategy, which can improve the efficiency of gene transfection in targeted tissues and drug delivery to organs [26, 27]. Using US microvesicles to carry therapeutic genes or drugs, UTMD acts on the target tissue, generating shear stress in cells to form transient cell membrane pores, improving cell membrane permeability [28] and promoting the targeted release of genes or drugs. This improves the efficacy of anti-tumour drugs and therapeutic genes [29] and reduces adverse reactions to drugs. Moreover, the effect of UTMD gene/drug therapy can be evaluated by observing the sonogram changes before and after disease treatment.

**Fig. 1:**
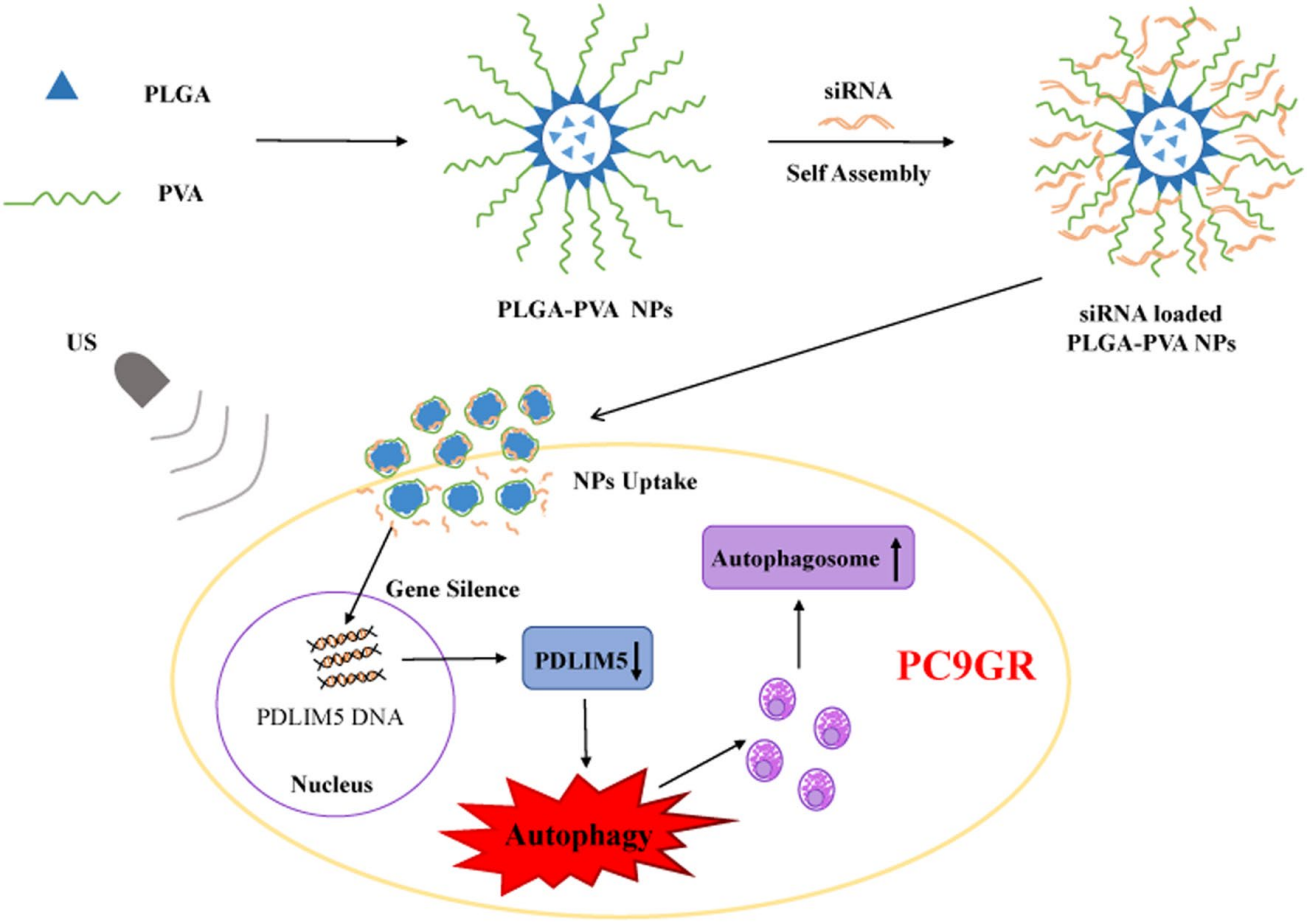
Graphical representation showing the formation of siRNA loaded PLGA-PVA NPs and autophagy in PC9GR cells. After the fabrication of siRNA loaded PLGA-PVA NPs, they enter into PC9GR cells with the coordination of US, and then suppress the expression of PDLIM5 gene and therefore induces autophagy which leads to the increase of autophagosomes. NPs, nanoparticles; US, ultrasound

This study investigates a different type of US sensitive siRNA-NB using the principle of positive and negative charge attraction by constructing an assembly of nanobubbles loaded with siRNA. This unique complex structure of siRNA-NBs can not only achieve high siRNA transfection efficiency under UTMD but also provide efficient siRNA protection. Moreover, given the recent discovery that PDLIM5 is an important cancer-related gene, with increased PDLIM5 expression levels in NSCLC associated with poor NSCLC prognosis, siRNA targeting PDLIM5 was chosen to evaluate the siRNA transfection of siRNA-NBs in PC9GR cells. It's complex optical and physical properties were determined using dynamic light scattering (DLS), transmission electron microscopy (TEM) and scanning electron microscopy (SEM). In vitro studies were further performed to evaluate its sensitivity to US and siRNA delivery capacity. Furthermore, its potential to enhance the sensitivity of NSCLC cells to EGFR-TKIs by targeting the *PDLIM5* gene and subsequently enhancing autophagy in PC9GR cells was determined. The experimental flow is shown in Fig. 1.

## Methods

### Materials and chemicals

PLGA-COOH (Shyuanye, Shanghai, China), dichloromethane (SCR, Shanghai, China), PVA (Sigma, USA), EDC (Ourchem, Shanghai, China), NHS (Shyuanye, Shanghai, China), PEI (1800 k) (Rhawn, Shanghai, China), RPMI-1640 medium and foetal bovine serum were purchased from GIBCO BRL (Grand Island, USA), Penicillin–Streptomycin Solution (C0222; Beyotime, Shanghai, China), Gefitinib (ZD1839; Selleck, USA), Lipo6000 Transfection Reagent(C0526FT; Beyotime, Shanghai, China), Cell Counting Kit-8(Yeasen, Shanghai, China), Hoechst 33342 (Solarbio, Beijing, China), Trizol Reagent Kit, Prime Script RT reagent Kit, 2× SYBR Green Pro Taq HS Premix reagent, ROX Reference Dye, SYBR Green Premix Pro Taq HS qPCR Kit and Evo M-MLV RT Kit with gDNA Clean for qPCR (Accurate Biotechnology, Hunan, China), RIPA Lysis Buffer(P0013B; Beyotime, Shanghai, China), Anti-PDLIM5/ENH antibody ab196559 (abcam, USA), GAPDH Rabbit Polyclonal antibody (Proteintech, USA), Peroxidase-Conjugated Goat anti-Rabbit IgG (H + L) (ORIGENE, Beijing, China), LC3B(D11)XP Rabbit mAb and SQSTM1/p62(D5E2) Rabbit mAb purchased from Cell Signaling (Massachusetts, USA).

Some siRNAs targeted gene PDLIM5 were designed from GenePharma (Shanghai, China), including PDLIM5-Homo-1782 (PDLIM5-1), PDLIM5-Home- 1375 (PDLIM5-2), PDLIM5-Homo-1048 (PDLIM5-3), and FAM labeled PDLIM5-Homo-1048, serial numbers shown in Table 1.

**Table 1:** Sequences of PDLIM5 siRNAs

ON-TARGET gene	Sense	Antisense
PDLIM5-1	5′-GGUACUAUAUGCCAUGGAUTT-3′	5′-AUCCAUGGCAUAUAGUACCTT-3′
PDLIM5-2	5′-GUGACCAGGACACUUUAGUTT-3′	5′-ACUAAAGUGUCCUGGUCACTT-3′
PDLIM5-3	5′-CUGGGACUGAACAUUUGAATT-3′	5′-UUCAAAUGUUCAGUCCCAGTT-3′

### Bioinformatic analysis of *PDLIM5*

The RNAseq data (level 3) and corresponding clinical information for NSCLC were obtained from The Cancer Genome Atlas (TCGA) database (https://portal.gdc.com). Log rank was used to compare the Kaplan–Meier survival analysis differences between normal group and NSCLC group. For the Kaplan–Meier curve, *P* values and hazard ratio (HR) with a 95% confidence interval (CI) were obtained using the log rank test and univariate Cox regression. The above analyses were performed using the v 4.0.3 version of the R software (R Foundation for Statistical Computing, 2020). *P* < 0.05 was considered statistically significant.

### Cells and culture conditions

Tyrosine kinase inhibitor (TKI)-sensitive LUAD cell line PC9 was purchased from Cellcook (Guangdong, China), and gefitinib-resistant LUAD cell line PC9GR was purchased from FUHENG BIOLOGY (Shanghai, China). PC9GR cells were routinely cultured in RPMI-1640 medium supplemented with 10% foetal bovine serum and 1% Penicillin–Streptomycin solution and incubated in 5% CO_2_ at 37 °C. Gefitinib was added to the culture medium at a concentration of 2 μmol/l to sustain the drug-resistance phenotype of PC9GR cells.

### Selection of siRNA and the expression of *PDLIM5* protein in PC9 and PC9GR cell lines

Specific ON-TARGET siRNAs were designed to silence *PDLIM5*. The sequences of *PDLIM5* are listed in Table 1. The effects of *PDLIM5* gene silencing were identified using western blot. ON-TARGET siRNAs were transfected into PC9GR cells using the Lipo6000 Transfection Reagent. The PC9GR cell line was divided into four groups: experimental groups were transfected with siRNAs targeted *PDLIM5* (PDLIM5-1, PDLIM5-2 and PDLIM5-3) and the control group was without any interference.

Moreover, *PDLIM5* protein expression levels in PC9 and PC9GR cells were compared to explore the correlation between *PDLIM5* and gefitinib resistance in PC9 cells.

### Formulation and synthesis of nanoparticles (NPs)

Briefly, 100 mg PLGA-COOH was directly dissolved in 4 ml dichloromethane. This mixture was then added into 20 ml 2% precooled PVA aqueous solution and homogenized. Under sonication, dichloromethane was removed. After high-speed centrifugation and DE precipitation, EDC, NHS, Milli-Q water and PEI were mixed and stirred. The NPs were collected via centrifugation and washed thrice with distilled water at the same parameters.

### The determination of the encapsulation efficiency of nanobubbles (NBs)

The siRNA solution was fully mixed with PLGA-NBs solution (1 mg/ml) in a 1:4 ratio. After centrifugation, phosphate-buffered solution and NBs solution were added to the precipitate. Subsequently, NBs carrying siRNA were obtained.

A spectrophotometer was used to evaluate the efficiency of the combination of siRNA and NBs. To evaluate the binding of siRNA to NBs during the above ligation process, unbound PDLIM5 siRNA in the above suspension was determined using a spectrophotometer (Thermo ND2000, Thermo Science Company, USA). The experiment was performed in triplicates. Encapsulation efficiency of NBs (EE, %) = (total amount of siRNA in NB suspension-the total amount of unbound siRNA)/total amount of siRNA in NB suspension.

### Material characterisation of NPs

#### Observation of particle size, polymer dispersity index (PDI), zeta potentials and NP morphology

A Malvern Nano ZS detector (Malvern Instruments, Malvern, England) was used to measure the particle size, PDI and zeta potential of NPs according to the principle of DLS. The reported NP formulation values are presented as mean ± standard deviation of the lowest three individual measurements for each sample.

The NP morphology was investigated using TEM and SEM. For TEM, the NP suspension was deposited on a copper grid and dried in a desiccator. Subsequently, the morphology of each group of the NPs was observed under HT7800 TEM (Hitachi, Japan), and the images were acquired.

### Stability and US sensitivity tests

To compare the stability of siRNA-NBs and NBs with traditional SonoVue Microbubbles (MBs), in vitro contrast enhanced US imaging experiments were performed using a custom made 3% (w/v) agarose mould. A total of 1 ml NBs or siRNA-NBs with a bubble concentration of 3 × 10^6^ bubbles/ml was added to the sample wells. A high-frequency linear transducer of the clinical US scanner system (Philips EPIQ7, Philips, USA) was set to a frequency of 20 MHz, an ultrasound intensity of 4% and a gain of 30 dB. Three ultrasonic images were recorded for each sample in the initial and at 40 min for the off line gray-scale intensity examinations using the ImageJ software.

### Cytotoxicity analysis of NPs

Cell viability was assessed using the Cell Counting Kit-8 (CCK8) assay (Yeasen, Shanghai, China), following the manufacturer’s suggestions. Briefly, the PC9GR cells transfected with NB-siRNA (untreated PC9GR cells were included as negative controls) were cultivated in five 96-well plates with six replicate wells, followed by incubation in a humidified incubator for 3 h. A total of 10 μl CCK-8 solution was added to each well, incubated for 2 h and assayed using a microplate reader at 450 nm.

### Transfection efficiency measurements

To investigate the transfection efficiency of the NPs, confocal laser microscope observation (CLSM, Zeiss LSM880, Carl Zeiss, Germany) was performed. Briefly, PC9GR cells were seeded on a confocal dish. The experimental group cells were transfected with siRNA, siRNA-NBs, siRNA + US and siRNA-NBs + US. The following conditions were applied for UTMD: ultrasound intensity: 500 Mw/DM^2^; duty cycle: 20%; pulse rate1000 Hz, irradiation duration of 90 s. All siRNA were FAM-labelled PDLIM5 siRNA. Untreated PC9GR cells were included as negative controls. The nucleus was stained with Hoechst 33342 and cells were observed using a confocal laser scanning microscope.

### Effect of autophagy in PC9GR cells

The effect of autophagy in PC9GR cells was analysed via autophagosome formation observed using TEM (HT7800, Japan), and autophagy-related proteins (p62 and LC3-II/I) were measured using western blot.

### Molecular assays

#### Real-time polymerase chain reaction (PCR) assay for determining the mRNA level of *PDLIM5*

Total RNA was harvested from the cell using the Trizol Reagent Kit, according to the manufacturer’s protocol. RNA yield was determined using a NanoDrop spectrophotometer (Thermo Fisher, Waltham, USA). Real-time PCR was performed using TB Green qPCR Mix Plus (Accurate Biotechnology, Hunan, China) and the CFX96TM Real-time Detection System (Accurate Biotechnology, Hunan, China). Glyceraldehyde 3-phosphate dehydrogenase (GAPDH) was used as an endogenous reference. Data were analysed using the relative standard curve method, according to the manufacturer’s protocol. All data were normalised against GAPDH mRNA levels and expressed as fold increases relative to controls. The primer sequences of the tested genes are listed in Table 2.

**Table 2:** Primers used in this study

Primers	Sequences (5′–3′)
PDLIM5-F	GCAGCCCAGGCAAATGTAAG
PDLIM5-R	CACAGAACCAAAAGGCCGTG
GAPDH-F	GGAGTCCACTGGCGTCTTCA
GAPDH-R	GTCATGAGTCCTTCCACGATACC

### Western blot analysis

Total protein was extracted and quantified using RIPA Lysis Buffer and bicine cholinic acid protein assay kit, respectively. Protein samples were separated on a 10% or 15% sodium dodecyl sulfate–polyacrylamide gel electrophoresis (SDS-PAGE) and then transferred to polyvinylidene difluoride (PVDF) membranes. After incubation with the blocking buffer, the membranes were incubated overnight at 4℃ with rabbit antibodies against PDLIM5 (1:1000 dilution), LC3 (1:1000 dilution) and P62 (1:1000 dilution). Simultaneously, the membranes were incubated with rabbit antibodies against GAPDH (1:25,000 dilution) as an internal standard for normalizing protein expressions. Peroxidase-Conjugated Goat anti-Rabbit IgG (H + L) (1:25,000 dilution) was used to amplify the signal. Protein signals were detected using a chemiluminescence system (Tanon 5200Uulti, Shanghai, China).

### Statistical analyses

The data were statistically analysed using a one-factor analysis of variance (SPSS software, version 24.0, SPSS Inc. and GraphPad Prism 8). All data are expressed as the mean ± standard errors of the mean. *P* < 0.05 was considered statistically significant. All experiments were performed in triplicates.

## Results

### Bioinformatic analysis of *PDLIM5*

**Fig. 2:**
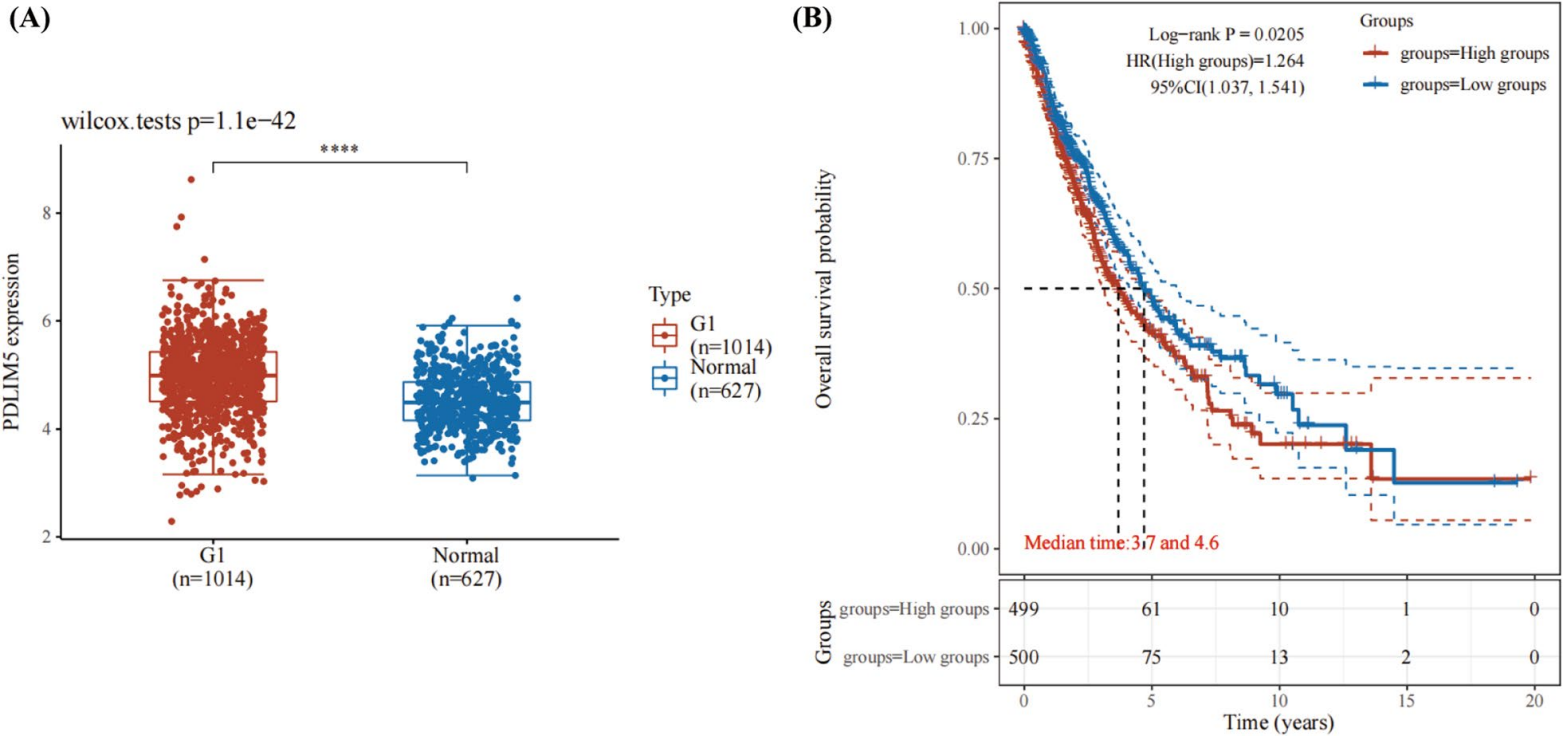
Bioinformatic analysis of *PDLIM5*. **A** The expression distribution of *PDLIM5* in non-small cell lung cancer (NSCLC) tissues (Red colour: G1 group) and normal tissues (Blue colour: Normal group). The abscissa represents the different groups of samples, and the ordinate represents the expression distribution of the gene. *Represents the significant *P* values, ****P < 0.001. The statistical difference between the two groups was compared using the Wilcox test. **B** Overall survival probability in different groups with a high or low expression of *PDLIM5*. Red: high *PDLIM5* expression, Blue: low *PDLIM5* expression. Kaplan–Meier survival analysis of the gene signature from The Cancer Genome Atlas dataset and different groups were compared using a log-rank test. Hazard Ratio (HR) (High exp) represents the HR of the low-expression sample compared to the high-expression sample. HR > 1 indicates a risk factor. The median survival time (LT50) for the high and low expression groups are 3.7 and 4.6 years, respectively

The RNAseq data (level 3) of NSCLC samples and the corresponding clinically informative expression distribution of *PDLIM5* in tumour and normal tissues from TCGA were obtained (https://portal.gdc.com). *PDLIM5* expression in NSCLC tissues was significantly increased compared with normal tissues (*P* < 0.001) (Fig. 2A); Following this, NSCLC samples were divided into high or low expression *PDLIM5* groups (Fig. 2B). The viability in each group was analysed using the log-rank test. HR (high exp) indicates the hazard ratio of low expression samples relative to high expression samples. HR > 1 was considered a risk factor indication. The median survival time (LT50) in the high and low expression *PDLIM5* groups was 3.7 and 4.6 years, respectively, indicating that the level of *PDLIM5* expression is inversely proportional to the survival time.

### Screening of siRNA targeted PDLIM5 and the expression of PDLIM5 in PC9 and PC9GR cells

**Fig. 3:**
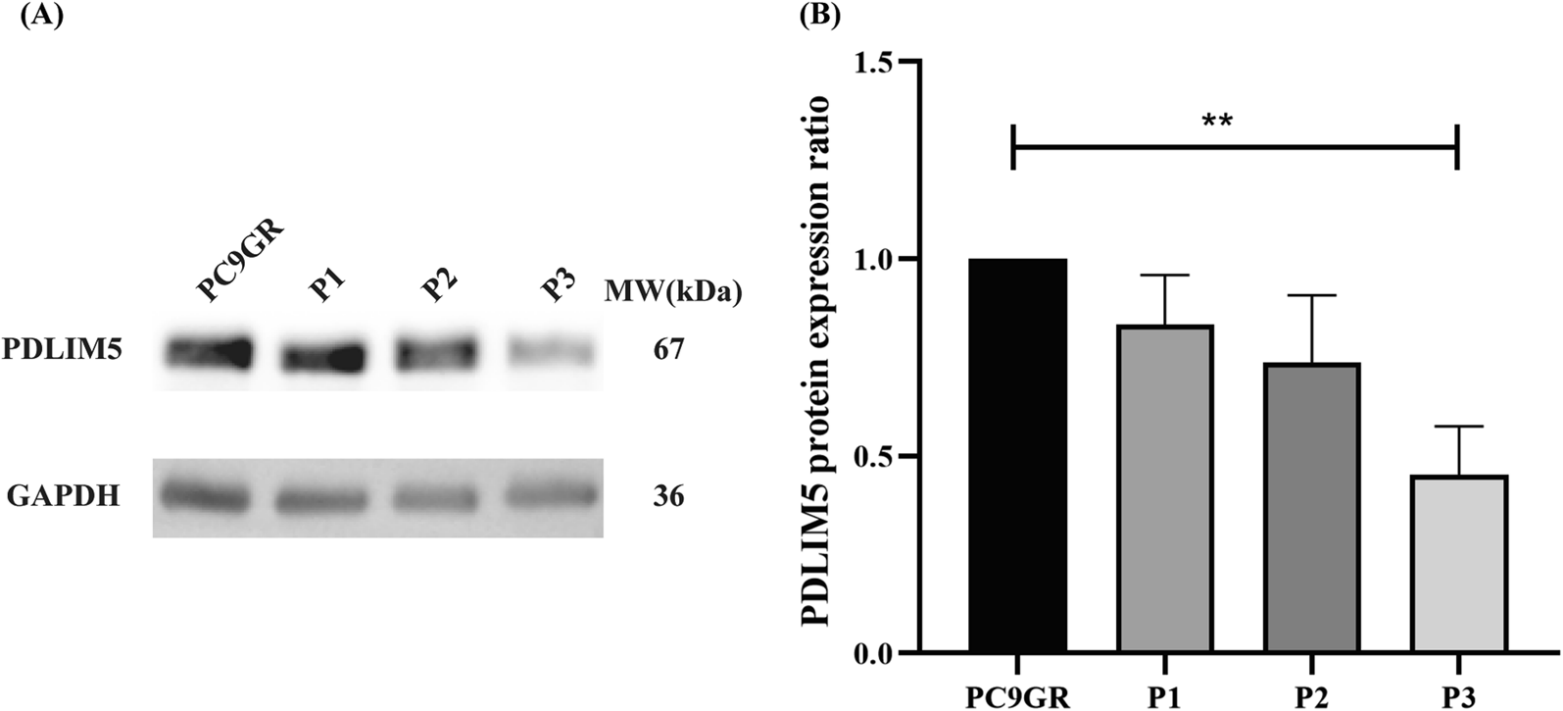
Expression of *PDLIM5* in PC9GR cells. **A** and **B** Western blot analysis of PDLIM5 protein levels in PC9GR cells transfected with different siRNAs (P1: PDLIM5-1, P2: PDLIM5-2, P3: PDLIM5-3) for 48 h, GAPDH served as an internal control. Compared with the control group (PC9GR), the expression of PDLIM5 decreased in the P3 group and had a significant difference (n = 3) (**P < 0.005)

To screen for siRNA targeted PDLIM5, the role of PDLIM5 gene suppression in PC9GR cells after transfection with different siRNAs was investigated. As shown in Fig. 3, the protein of PDLIM5/GAPDH level in the PDLIM5-3 (P3) group significantly reduced than in the PDLIM5-1 (P1) and PDLIM5-2 (P2) groups compared with the control group (n = 3) (P3: 0.55 ± 0.09, *P* < 0.005; P1: 1.06 ± 0.31, *P* > 0.05 and P2: 0.95 ± 0.34, *P* > 0.05, respectively). This indicates that the siRNA (PDLIM5-3) can effectively silence PDLIM5.

**Fig. 4:**
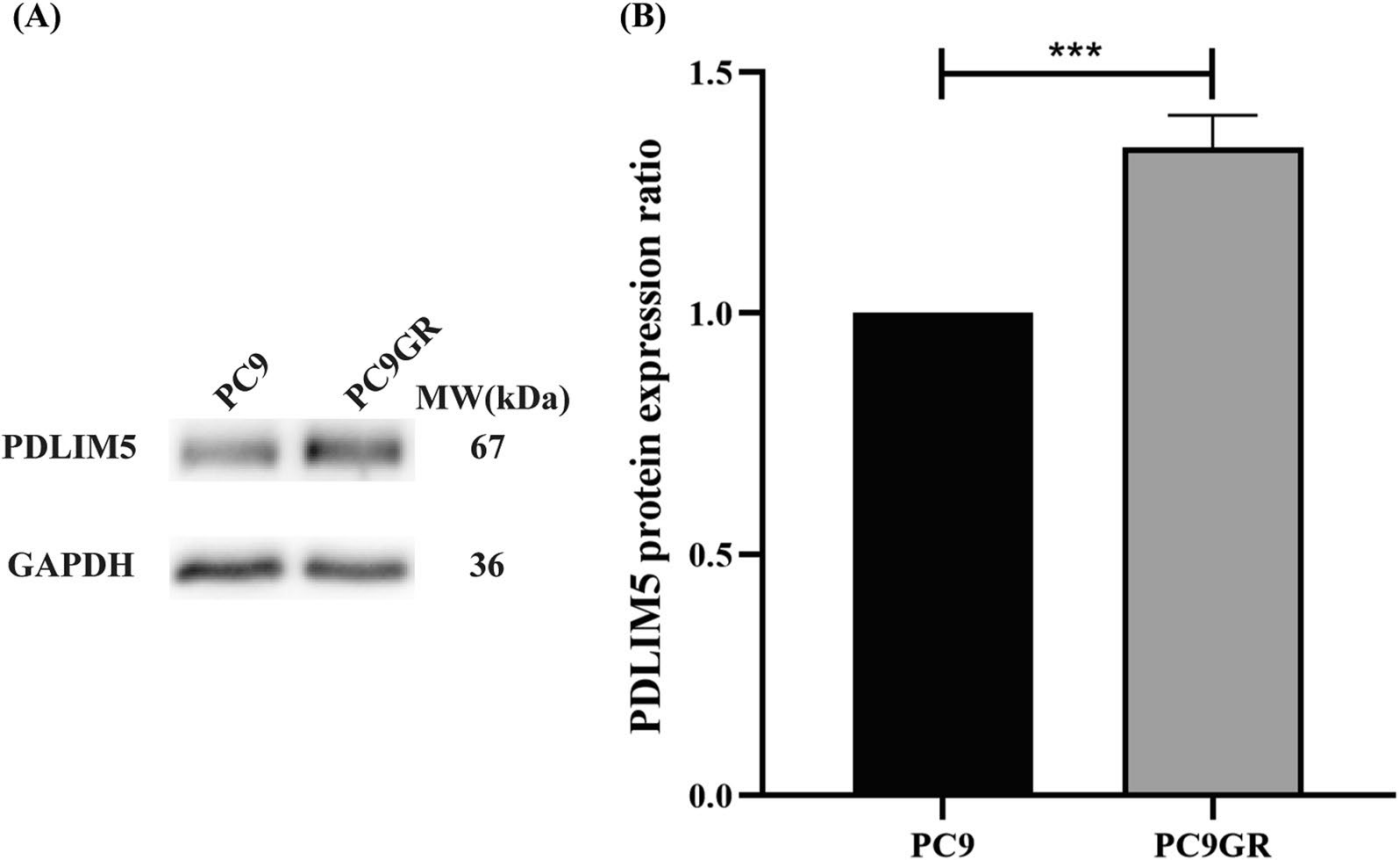
Expression of PDLIM5 gene in PC9 and PC9GR cells. **A** and **B** Western blot analysis of PDLIM5 protein levels in PC9 and PC9GR cells for 48 h, Glyceraldehyde 3-phosphate dehydrogenase served as an internal control. Compared with the PC9 group, the expression of PDLIM5 increased in the PC9GR group (n = 3). (***P < 0.001)

Furthermore, the analysis of protein PDLIM5 expression in PC9 and PC9GR cells revealed that protein of PDLIM5/GAPDH level increased in PC9GR cells compared to that in PC9 cells (n = 3) (0.85 ± 0.11 *vs* 0.63 ± 0.05, *P* < 0.0001, Fig. 4). Thus, the level of PDLIM5 expression could be associated with gefitinib resistance in PC9 cells, even playing a promoting role in gefitinib resistance.

### Material characterisation of NPs

**Fig. 5:**
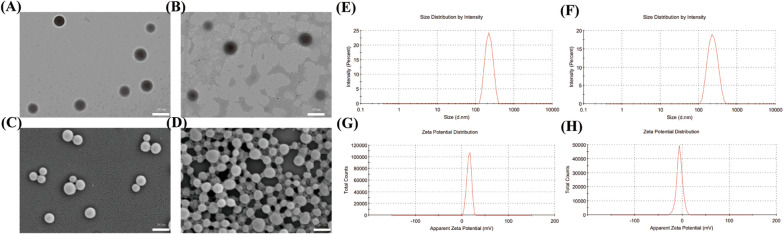
Material characterisation of Nanoparticles (NPs). **A** and **B** Transmission Electron Microscopy images of nanobubbles (NBs) and ultrasound sensitive siRNA-loaded nanobubbles (siRNA-NBs) (scale: 200 nm); **C** and **D** Scanning electron microscopy images of NBs and siRNA -NBs (scale: 200 nm). **E** and **F** The average sizes of NBs and siRNA-NBs were 216.30 ± 1.54 nm and 223.17 ± 2.23 nm, respectively; **G** and **H** The average zeta potentials of NBs and siRNA -NBs are 22.63 ± 0.55 mV and -1.94 ± 3.72 mV, respectively

TEM and SEM images showed homogeneous bubble sizes among the NBs and siRNA-NBs, with well-defined spherical morphology, smooth surface, high dispersity, no aggregation (Fig. 5A–D) and particle size distribution ranging from 200 to 250 nm. Additionally, DLS showed similar results. The average sizes of NBs and siRNA-NBs were 216.30 ± 1.54 nm and 223.17 ± 2.23 nm (n = 3), and a slight size increase was observed (Fig. 5E, F and Table 3); The average zeta potentials of NBs and siRNA-NBs are 22.63 ± 0.55 mV and − 1.94 ± 3.72 mV, n = 3 (Fig. 5G, H and Table 3).

**Table 3:** Characterization of the different formulations of NBs

Groups	Size distribution(nm)	PDI	Zeta potential(mV)
Pure NBs	216.30 ± 1.54	0.07	22.63 ± 0.55
siRNA-NBs	223.17 ± 2.23	0.08	− 1.94 ± 3.72

The mean particle size of siRNA-NBs was slightly larger than that of NBs, indicating that the presence of siRNA enlarged the volume of the NBs (*P* < 0.05). Owing to the particle size (200–250 nm), they can enter the tumour tissue gap and play the role of a therapeutic and US contrast agent, thereby allowing the real-time dynamic observation of the treatment effect.

### In vitro assessment of target binding

The strategy of combining siRNA with NBs was validated by the EE of NBs, wherein EE = 94.08 ± 0.28%, n = 3. This indicated that a good connection between PDLIM5 siRNA and NBs could be achieved through the principle of positive and negative charge attraction.

### Stability and US sensitivity tests

**Fig. 6:**
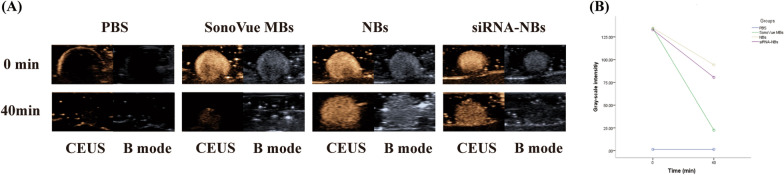
The stability of SonoVue MBs, nanobubbles (NBs) and ultrasound (US) sensitive siRNA-loaded nanobubbles (siRNA-NBs). Stability of SonoVue MBs, NBs and siRNA-NBs analysed using contrast enhanced US imaging in vitro (**A**). At the initial stage (0 min), there was no significant difference in the gray-scale intensity among the SonoVue MBs, NBs and siRNA -NBs groups, indicating that NBs and siRNA-NBs did not change the US sensitivity. After 40 min, compared with SonoVue MBs, the gray-scale intensities of NBs and siRNA-NBs declined more slowly, confirming their strengthened stability (**B**)

Representative contrast enhanced US images until 40 min after US exposure are shown in Fig. 6. There were no differences of gray-scale intensities among SonoVue MBs, NBs and siRNA-NBs at the initial stage (0 min) (134.2 ± 2.52, 132.85 ± 2.52 and 134.70 ± 2.37, *P* > 0.05, n = 3, respectively), indicating that NBs and siRNA-NBs did not change the US sensitivity. However, after 40 min, the gray-scale intensity of SonoVue MBs reduced more significantly than that of the NBs and siRNA-NBs (22.53 ± 0.68 *vs* 80.50 ± 0.81 and 94.32 ± 3.26, *P* < 0.0001, n = 3), and there was no differences between NBs and siRNA-NBs (80.50 ± 0.81 *vs* 94.32 ± 3.26, *P* > 0.05, n = 3), indicating that NBs and siRNA-NBs were structurally more stable than SonoVue MBs.

### Cytotoxicity analysis of NPs

**Fig. 7:**
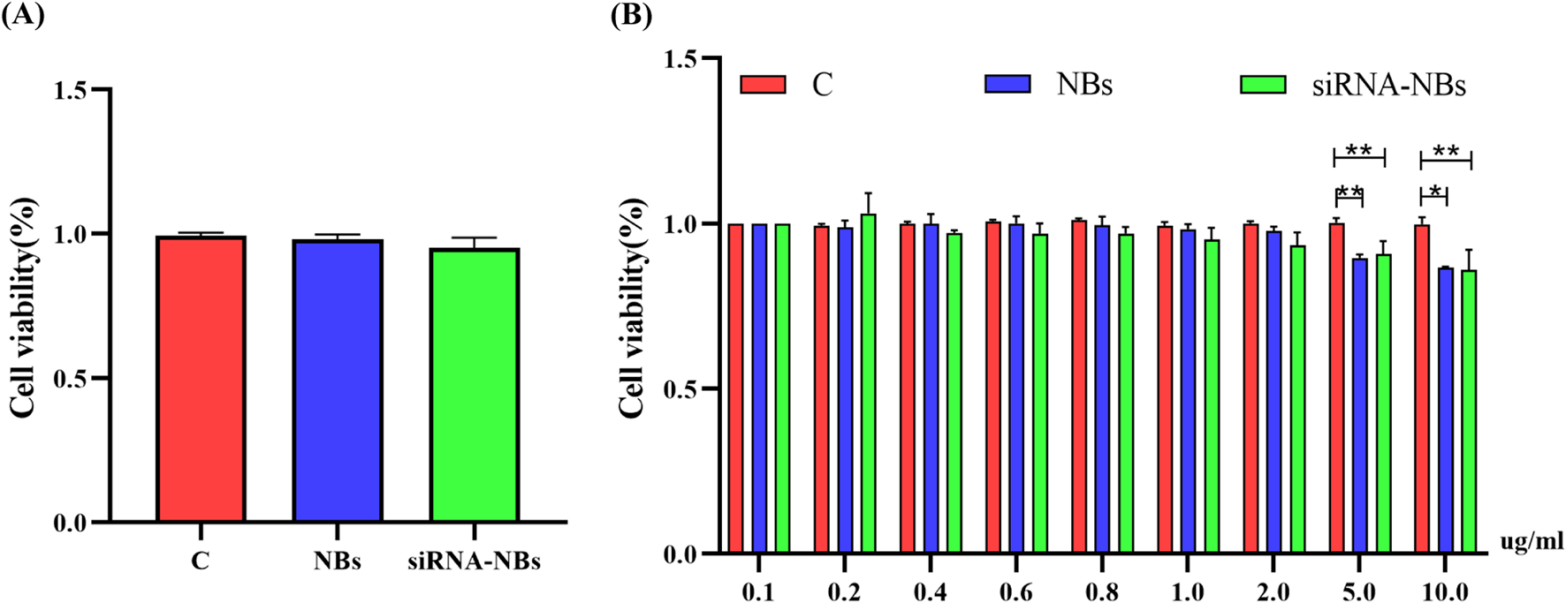
The cytotoxicity of NBs and US sensitive siRNA-NBs in PC9GR cells was determined using a CCK-8 assay. As shown in Fig. 5A, the control, NB and siRNA -NB groups showed no obvious cytotoxicity, and the cell viability rate was above 95%. In Fig. 5B, there was no significant difference in cell viability rate among the three groups when the concentration varied from 0.1 to 2.0 ug/ml (*P* > 0.05). However, compared with the control group, the cell viabilities of the NB and siRNA-NBs groups were significantly reduced (*P* < 0. 05) up to 5 ug/ml, but they remained more than 75% viable

The viability of the increasing concentration of NPs in PC9GR cells was detected using a CCK-8 assay. The cells viability obtained were (98.82 ± 1.19)%, (94.39 ± 2.88) % and (96.77 ± 1.18) % for control, NBs and siRNA-NBs groups respectively, and there were no significant differences among these groups (*P* < 0.05, n = 3), indicating that NBs and siRNA-NBs did not trigger significant cytotoxicity, (Fig. 7A). Moreover, there was no significant difference in cell viability rate when the concentration varied from 0.1 to 2.0 ug/ml among the three groups (*P* > 0.05). However, compared with the control group, the cell viabilities of the NBs and siRNA-NBs groups were significantly reduced (*P* < 0.05) until the concentration of 5 ug/ml (Fig. 7B). The siRNA-NBs showed dose-dependent cytotoxicity.

### Transfection efficiency assay

**Fig. 8:**
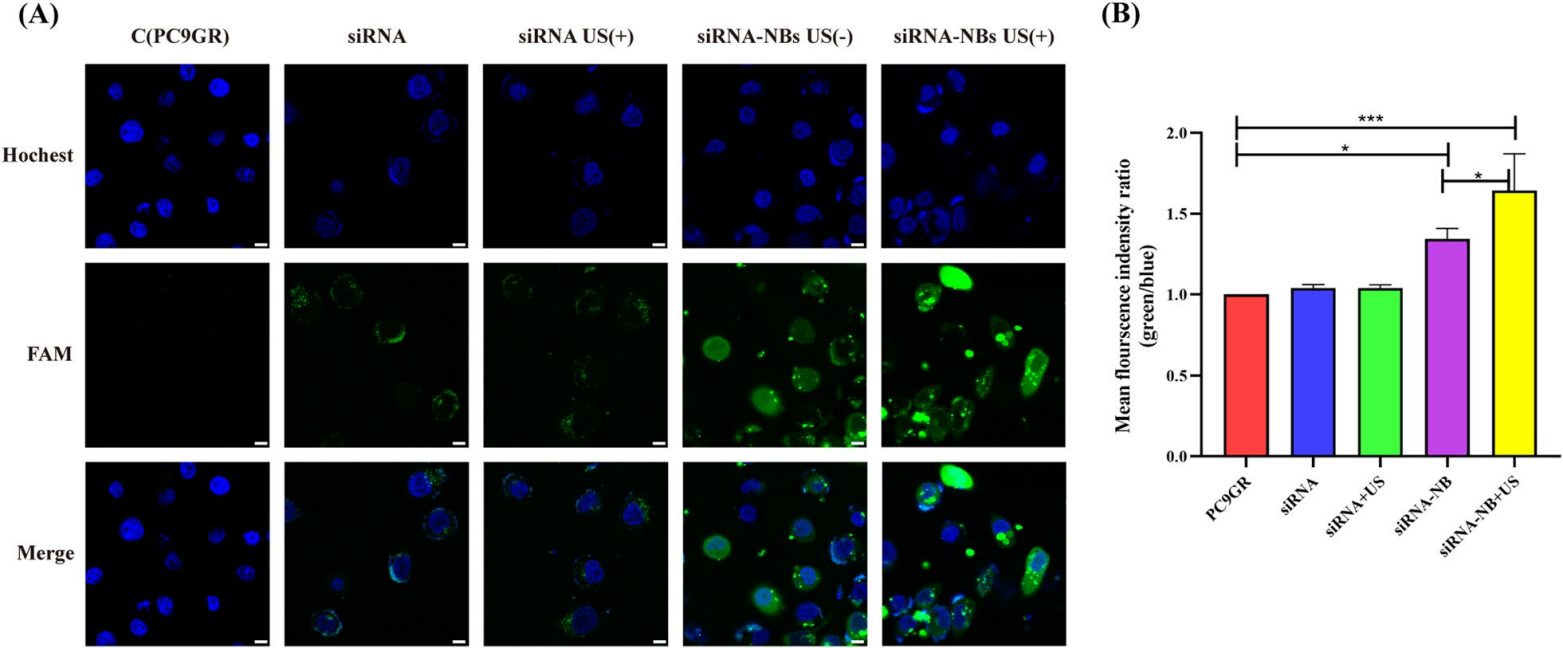
Transfection efficiency assay. **A** Laser confocal microscopic images (64×) of PC9GR cells after being transfected. More intense green fluorescence was observed in the cytoplasm and the periphery of nuclei in the siRNA -NBs US (±) groups. **B** Mean fluorescence intensity ratio (green/blue) of each group. The mean fluorescence intensity ratio was much higher in the siRNA -NBs US (±) groups, with the siRNA-NBs US (+) group showing the most effective increase. (*P < 0.05,***P < 0.001). siRNA-NBs, siRNA-loaded nanobubbles; US, ultrasound (Scale:10 um)

As shown in Fig. 8, compared with control group, the FAM fluorescence intensity of cells in the siRNA-NBs and siRNA-NBs + US groups were 5.05 ± 0.68 and 9.48 ± 0.68, which were higher than that of other groups after transfection (siRNA: 0.98 ± 0.17 and siRNA + US: 0.98 ± 0.17, *P* < 0.01, n = 3), with the siRNA-NBs + US group showing the most effective increase (9.48 ± 0.68 *vs* 5.05 ± 0.68, *P* < 0.001, n = 3). The weakest FAM fluorescence was observed in the control group and a mild increase was observed in the siRNA and siRNA + US groups. Notably, there was no significant difference among the groups (*P* > 0.05). This observation is consistent with a previous study reporting that nanocomposite delivery enhanced the cell update of fluorescence. Importantly, the combination of nanocomposite delivery and UTMD increased the intracellular delivery of siRNA, thus improving the transfection efficiency.

### Suppression of *PDLIM5* expression

**Fig. 9:**
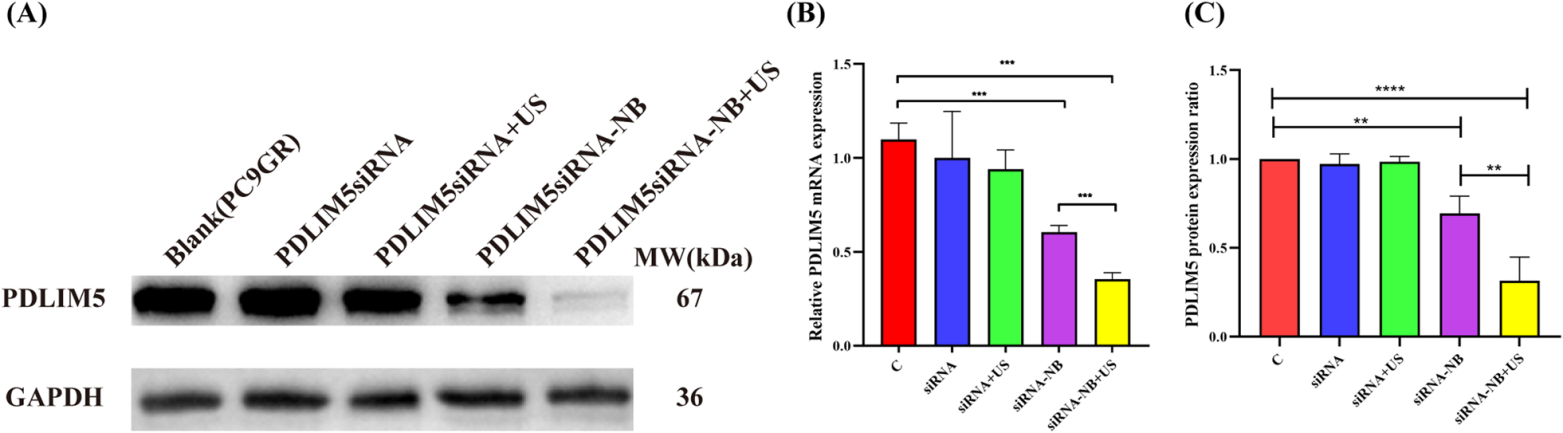
Expression of *PDLIM5* in PC9GR cells. PDLIM5 expression was analysed at the protein level using western blotting (**A**). The expression of *PDLIM5* in PC9GR cells was evaluated at the mRNA level using a real-time polymerase chain reaction (**B**). The expression of *PDLIM5* was increased in the siRNA-NB and siRNA-NB + US groups, with the siRNA-NB + US group showing the most effective increase (**C**) (*P < 0.05, **P < 0.01, ***P < 0.001, ****P < 0.0001)

Compared with the negative control group, the PDLIM5 level decreased in siRNA-NBs and siRNA-NBs + US groups. Moreover, US exposure played a key role in further decreasing the PDLIM5 level (Fig. 9A). As shown in Fig. 9B, the PDLIM5 mRNA level decreased significantly in siRNA-NBs and siRNA-NBs + US groups than that in siRNA and siRNA + US groups (0.60 ± 0.04, 0.35 ± 0.04 *vs* 1.00 ± 0.09, 0.94 ± 0.10, *P* < 0.001, n = 3), with the most decrease in siRNA-NBs + US group compared with siRNA-NBs group (*P* < 0.001). The expression of PDLIM5 at the protein level was further determined using western blotting (Fig. 9C), compared with the control group, the protein of PDLIM5/GAPDH level, were 0.69 ± 0.10 and 0.31 ± 0.13 in both siRNA-NBs and siRNA-NBs + US groups, which significantly reduced than that in siRNA and siRNA + US groups (0.97 ± 0.06 and 0.98 ± 0.03) (*P* < 0.01, n = 3), with the siRNA-NBs + US group showing a significant decrease (*P* < 0.0001).

### Suppression of *PDLIM5* gene expression induces autophagy in PC9GR cells

**Fig. 10:**
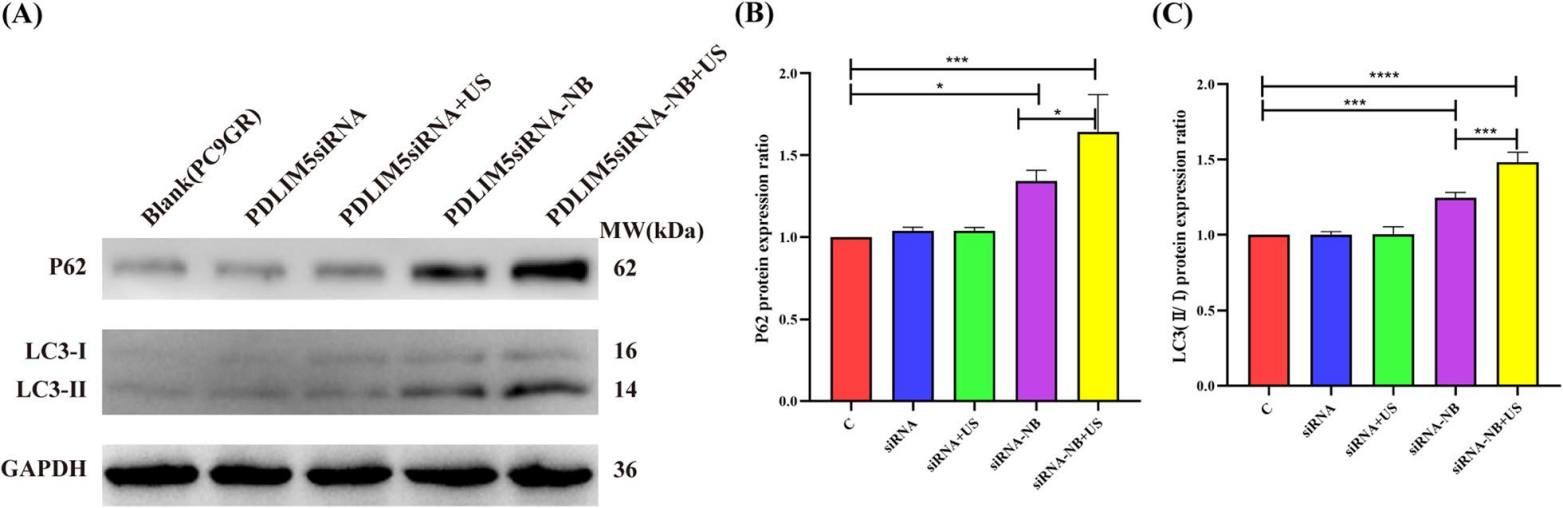
Suppression of *PDLIM5* expression induces autophagy in PC9GR cells. P62, LC3I and LC3II expressions at the protein level using western blotting (**A**). The expression of P62 and LC3II/I proteins was increased in the siRNA -NB and siRNA-NB + US groups, with the siRNA-NB + US group showing the most effective increase (**B**, **C**). (*P < 0.05,***P < 0.001, ****P < 0.0001)

**Fig. 11:**
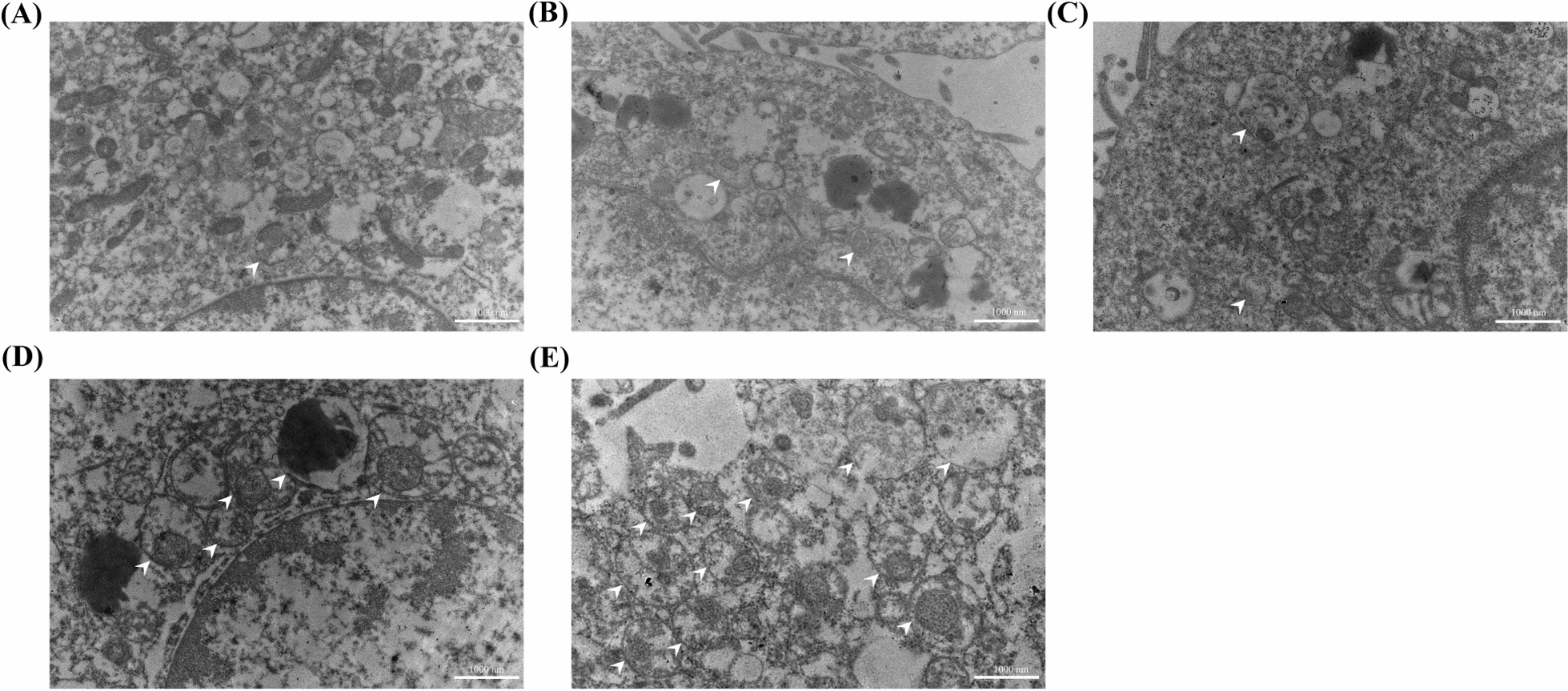
Autophagosomes in PC9GR cells after transfection under a transmission electron microscope. A large number of autolysosomes/autophagosomes (white arrows) were observed per cellular cross-section in the PDLIM5-NBs US (±) groups (**D**, **E**) compared to other groups (**A**–**C**) with the PDLIM5-NBs + US group showing the most number of autophagosomes (Scale: 1000 nm)

Further, the role of *PDLIM5* gene suppression in the regulation of autophagy in PC9GR cells after transfection with NBs was investigated. As shown in Fig. 10A, the expression of LC3-II/I and p62 increased in siRNA-NBs and siRNA-NBs + US groups compared to other groups. The protein of P62/GAPDH level in siRNA-NBs and siRNA-NBs + US groups significantly increased than that in siRNA and siRNA + US groups (1.34 ± 0.06, 1.64 ± 0.23 *vs* 1.04 ± 0.02, 1.04 ± 0.02, *P* < 0.05, n = 3), with the most decrease in siRNA-NBs + US group compared with siRNA-NBs group (*P* < 0.05) (Fig. 10B). The protein of LC3-II/I level in siRNA-NBs and siRNA-NBs + US groups significantly increased than that in siRNA and siRNA + US groups (1.25 ± 0.04, 1.48 ± 0.07 *vs* 1.00 ± 0.02, 1.00 ± 0.05, *P* < 0.001, n = 3), with the most decrease in siRNA-NBs + US group compared with siRNA-NBs group (*P* < 0.001) (Fig. 10C). To further clarify the effect of autophagy in the suppression of *PDLIM5* gene expression in PC9GR cells, autophagosomes in the cross-sections of PC9GR cells after transfection were determined using TEM. The siRNA-NBs US ( ±) groups showed higher numbers of autophagosomes per cellular cross-section compared to other group cells (Fig. 11). Therefore, the suppression of *PDLIM5* expression induces autophagy.

## Discussion

UTMD-mediated drug or gene delivery system has the following advantages: (1) safe; (2) non-invasive; (3) therapeutic effects of drug and microbubble delivered via US imaging and/or gene, integrating the diagnosis and treatment of target tissues; (4) Economic and practical; (5) strong targeted delivery ability; and (6) longer stay in the tumour site [26, 30].

Compared with traditional US contrast agents, NBs have a smaller size, higher stability and longer cycle time [31]. In this study, NPs with particle sizes around 200 nm were developed that not only have the above characteristics but also can penetrate tumour tissue more efficiently. This is attributed to the fact that the endothelial gap ranges from 380 to 780 nm [32] while microbubbles of size 1–10 mm usually cannot leak from the blood vessels into the tumour tissue [33]. Based on these features, UTMD technology and NBs were combined to deliver siRNA into drug resistant-tumour cells, thereby exploring the correlation between them.

The role of tumour cell autophagy in chemotherapy could be associated with the tumour type, stage, chemotherapy type and tumour cell bearing capacity. Autophagy is a double-edged sword that determines the final fate of tumour cells. Mild autophagy could reduce the sensitivity of tumour cells to chemotherapeutic drugs while intense or prolonged autophagy could promote tumour cell resistance. Therefore, the exact mechanism of autophagy on chemotherapeutic resistance in tumour cells needs further investigation.

Various studies have confirmed that LC3-II is necessary for the formation of autophagosomes and corresponds to the number of autophagosomes [34]. Therefore, LC3-II is generally regarded as a marker of mammalian autophagosomes [35]. Similarly, after the silencing of *PDLIM5* in PC9GR cells, the number of autophagosomes increased with the increase in LC3-II expression. Additionally, studies have confirmed that protein P62 has a special domain called the microtubule-associated protein light chain 3 interaction region (LIR). The LIR domain is required for autophagy degradation, which ensures the targeted binding of autophagy receptors to the microtubule-associated protein light chain 3 (LC3) anchored in the autophagosome membrane [35–38]. The LIR domain interacts with LC3 to promote autophagosome formation and deliver the polyubiquitinated “cargo” for autophagy [39]. Hence, during autophagy formation, LC3-II and P62 are speculated to not show a syntropy change. However, the same syntropy was found in this study.

Additionally, a few studies have shown an increase in the number of P62 in various cancer cell types, including lung cancer [35, 40], which could also be attributed to the abnormally elevated P62 expression in PC9GR cells. Despite autophagy being enhanced after the inhibition of *PDLIM5* in PC9GR cells, the reduction of P62 expression could be less than the abnormally increased expression in PC9GR cells, so not only the increasement of the expression of LC3-II, but also P62 increased as the same well. As P62 is a hub between autophagy defects and key pathways regulating carcinogenesis, such as inflammation, redox homeostasis and (energy) metabolism [41], it participates in the metabolic support of tumour growth and contributes to tumour drug resistance [42]. As a result of *PDLIM5* silencing in PC9GR cells, P62 is speculated to be involved in multiple tumour related pathways; however, overall P62 expression was increased in the PC9GR cells. Hence, further studies are required to validate the increased expression of LC3-II and P62 during autophagy.

Additionally, the number of autophagy-related protein LC3II and autophagosome increased in PC9GR cells after *PDLIM5* silencing, which indicated that autophagy could be correlated with the sensitivity to gefitinib and *PDLIM5* could be involved in the resistance to gefitinib in PC9GR cells.

## Conclusion

Chemotherapeutic resistance observed in the treatment of NSCLC is the key factor restricting therapeutic efficacy. This study elucidates the PDLIM5 genes associated with NSCLC resistance and associated autophagic mechanisms. It showed that PDLIM5 played a role in the autophagy-mediated resistance in gefitinib-resistant PC9GR cells. Moreover, with the application of UTMD and a US-sensitive nanocarrier siRNA-NBs, siRNA can be effectively delivered into tumour cells for drug resistance studies. Using UTMD, US-sensitive siRNA-NBs have the potential as an ideal delivery vector to mediate highly effective RNA interference for NSCLC cells. This mode of administration overcomes the limitations of large particle size and insufficient siRNA loading seen in traditional MBs. Therefore, this study provides novel insights and directions in the treatment of chemotherapy resistance in PC9GR cells.

## Data Availability

The original contributions presented in the study are included in the article/additional files. Further inquiries can be directed to the corresponding authors. The data and materials used in the current study are available from the corresponding author on reasonable request.

## Abbreviations

UTMD: Ultrasound-targeted microbubble destruction
US: Ultrasound
siRNA-NBs: SiRNA-loaded nanobubbles
EGFR-TKIs: Epidermal growth factor tyrosine kinase inhibitors
NSCLC: Non-small cell lung cancer
LUAD: Lung adenocarcinoma
ENH: Enigma homolog
EGFR: Epidermal growth factor receptor
DLS: Dynamic light scattering
TEM: Transmission electron microscopy
SEM: Scanning electron microscopy
HR: Hazard ratio
CI: Confidence interval
TKI: Tyrosine kinase inhibitor
NPs: Nanoparticles
NBs: Nanobubbles
EE: Encapsulation efficiency
PDI: Polymer dispersity index
MBs: Microbubbles
PCR: Polymerase chain reaction
GAPDH: Glyceraldehyde 3-phosphate dehydrogenase
SDS-PAGE: Sodium dodecyl sulfate–polyacrylamide gel electrophoresis
PVDF: Polyvinylidene difluoride
LIR: Light chain 3 interaction region
